# Patient Reactions to Artificial Intelligence–Clinician Discrepancies: Web-Based Randomized Experiment

**DOI:** 10.2196/68823

**Published:** 2025-05-22

**Authors:** Farrah Madanay, Laura S O'Donohue, Brian J Zikmund-Fisher

**Affiliations:** 1 Center for Bioethics and Social Sciences in Medicine University of Michigan–Ann Arbor Ann Arbor, MI United States; 2 Department of Radiology University of Michigan Medicine University of Michigan–Ann Arbor Ann Arbor, MI United States; 3 Health Behavior and Health Equity, Internal Medicine, Center for Bioethics and Social Sciences in Medicine University of Michigan–Ann Arbor Ann Arbor, MI United States

**Keywords:** artificial intelligence, medical maximizing-minimizing, early detection of cancer, decision making, radiologists, patient satisfaction, patient-physician relationship, communication

## Abstract

**Background:**

As the US Food and Drug Administration (FDA)–approved use of artificial intelligence (AI) for medical imaging rises, radiologists are increasingly integrating AI into their clinical practices. In lung cancer screening, diagnostic AI offers a second set of eyes with the potential to detect cancer earlier than human radiologists. Despite AI’s promise, a potential problem with its integration is the erosion of patient confidence in clinician expertise when there is a discrepancy between the radiologist’s and the AI’s interpretation of the imaging findings.

**Objective:**

We examined how discrepancies between AI-derived recommendations and radiologists’ recommendations affect patients’ agreement with radiologists’ recommendations and satisfaction with their radiologists. We also analyzed how patients’ medical maximizing-minimizing preferences moderate these relationships.

**Methods:**

We conducted a randomized, between-subjects experiment with 1606 US adult participants. Assuming the role of patients, participants imagined undergoing a low-dose computerized tomography scan for lung cancer screening and receiving results and recommendations from (1) a radiologist only, (2) AI and a radiologist in agreement, (3) a radiologist who recommended more testing than AI (ie, radiologist overcalled AI), or (4) a radiologist who recommended less testing than AI (ie, radiologist undercalled AI). Participants rated the radiologist on three criteria: agreement with the radiologist’s recommendation, how likely they would be to recommend the radiologist to family and friends, and how good of a provider they perceived the radiologist to be. We measured medical maximizing-minimizing preferences and categorized participants as maximizers (ie, those who seek aggressive intervention), minimizers (ie, those who prefer no or passive intervention), and neutrals (ie, those in the middle).

**Results:**

Participants’ agreement with the radiologist’s recommendation was significantly lower when the radiologist undercalled AI (mean 4.01, SE 0.07, *P*<.001) than in the other 3 conditions, with no significant differences among them (radiologist overcalled AI [mean 4.63, SE 0.06], agreed with AI [mean 4.55, SE 0.07], or had no AI [mean 4.57, SE 0.06]). Similarly, participants were least likely to recommend (*P*<.001) and positively rate (*P*<.001) the radiologist who undercalled AI, with no significant differences among the other conditions. Maximizers agreed with the radiologist who overcalled AI (β=0.82, SE 0.14; *P*<.001) and disagreed with the radiologist who undercalled AI (β=–0.47, SE 0.14; *P*=.001). However, whereas minimizers disagreed with the radiologist who overcalled AI (β=–0.43, SE 0.18, *P*=.02), they did not significantly agree with the radiologist who undercalled AI (β=0.14, SE 0.17, *P*=.41).

**Conclusions:**

Radiologists who recommend less testing than AI may face decreased patient confidence in their expertise, but they may not face this same penalty for giving more aggressive recommendations than AI. Patients’ reactions may depend in part on whether their general preferences to maximize or minimize align with the radiologists’ recommendations. Future research should test communication strategies for radiologists’ disclosure of AI discrepancies to patients.

## Introduction

Radiologists are trained to detect lung cancer from low-dose computerized tomography (LDCT) scans with high accuracy [1,2] and medical artificial intelligence (AI) promises to improve this accuracy detection by acting as a “second pair of eyes” [1,3,4]. As of August 2024, the US Food and Drug Administration (FDA) published a list of 950 authorized AI-enabled medical devices, 723 (76%) of which were in the field of radiology. The developers of a medical AI model meant for lung cancer screening, called Sybil, claim the AI can detect future lung cancer in CT scans up to 6 years before lesions become visible to human radiologists [5]. In a 2020 survey of American College of Radiology members, 33% of radiologists reported currently using AI, while 20% of those not currently using AI planned to use it in the next 1 to 5 years [6].

Despite this rise in medical AI among radiologists, patient acceptance lags. Previous research has found patients’ resistance to AI stems from beliefs that AI performs inferior to humans and cannot be held accountable for errors, lower objective and subjective understanding of AI versus human decision-making, and fear that AI does not meet patients’ unique needs [7-10].

In part due to concerns about AI accuracy, US federal policies emphasize including a “human in the loop” (HITL) when it comes to AI development and use. In 2021, the FDA, along with Health Canada and the United Kingdom’s Medicines and Healthcare Products Regulatory Agency, published 10 guiding principles for machine learning development [11]. One of the principles describes focusing on the performance of the “human-AI team,” with AI performing with a HITL rather than in isolation.

Despite these guidelines, little is known about how patients will respond to the HITL. Whereas previous research has focused on patients’ trust or mistrust of medical AI [7,8,10], few studies have examined patients’ trust in the clinician of the human-AI team. Research shows patients’ trust in their clinician is related to patient satisfaction, compliance, and health outcomes [12-14]. Yet, medical AI could fundamentally change the patient-clinician relationship, and erode patients’ trust in clinician expertise [15,16].

In this study, we inquired how human-AI discrepancies affect patients’ agreement with radiologist recommendations and satisfaction with their radiologist. We examined 2 directions of human-AI discrepancy. First, the radiologist can “overcall AI,” in which the radiologist identifies higher cancer risk than the AI and recommends more testing. Second, the radiologist can “undercall AI,” in which the radiologist identifies lower cancer risk than the AI and recommends less testing. We hypothesize that patients will least agree with the recommendation of the radiologist who undercalls AI.

We anticipate patients’ agreement with their radiologist’s recommendation will vary based on individual differences, like medical maximizing-minimizing (MMM) preferences. Whereas medical maximizers typically seek more aggressive and optional approaches to care, minimizers prefer no or passive medical intervention unless deemed completely necessary [17]. Researchers have shown that MMM is associated with preferences for cancer screening and surveillance [18,19], treatment of incidental findings on imaging tests [20], concern with stopping medication [21], pursuit of appropriate care [22], and avoidance of health care [23]. In this context, we hypothesize that measuring MMM might help to identify opportunities to tailor communication and guide shared decision-making about medical AI to patients with different underlying care preferences.

## Methods

### Participants and Procedure

This study was preregistered at Open Science Framework [24]. We programmed the web-based quantitative experimental questionnaire in Qualtrics (Silver Lake) and collected data in April and May 2024. A total of 1828 adult, English-speaking participants were recruited by Dynata [25]. Dynata facilitates data collection for research surveys through its panel of potential web-based participants. We provided Dynata with quotas for age, gender, and race and ethnicity based on the US population composition. Inclusion criteria were at least 18 years of age and residents of the United States. These quotas and inclusion criteria enabled us to receive a diverse range of participant reactions to our experiment.

### Study Design

The study was designed as a 1-way, between-subjects, randomized experiment. Participants assumed the role of a patient advised to undergo an LDCT scan for lung cancer screening. After reading the hypothetical scenario, participants were randomized to receive 1 of 4 results conditions, in which (1) a radiologist alone identified low risk of cancer and recommended a repeat screening CT in 6 months, (2) AI and a radiologist both identified low risk of cancer and recommended a repeat screening CT in 6 months, (3) AI identified medium risk of cancer and recommended immediate additional testing with a nuclear medicine examination (positron emission tomography–computed tomography [PET-CT]) but a radiologist identified low risk and recommended a repeat screening CT in 6 months (ie, radiologist undercalled AI), or (4) AI identified low risk of cancer and recommended a repeat screening CT in 6 months but a radiologist identified medium risk and recommended immediate additional testing (PET-CT; ie, radiologist overcalled AI). We used the randomizer within Qualtrics to randomly assign participants to conditions. Participants read the results both in an electronic health record (EHR) system report and in a transcript of an oral follow-up. Textbox 1 shows the LDCT scan results received in the 4 experimental conditions.

**Hypothetical results of the low-dose computed tomography scan, received by participants in the four experimental conditions.**

**Radiologist only**

- Impression:
  There is a 7-mm solid nodule in the left lung.
  Lung Imaging Reporting and Data System (Lung-RADS) 3: Probably benign. Low risk of malignancy (cancer) 1%-2%.
- Recommendation:
  Repeat low-dose computed tomography (LDCT) in 6 months to ensure no interval growth.

**Radiologist-AI (artificial intelligence) agreement**

- Impression
  LungDetect AI: There is a 7-mm solid nodule in the left lung. Lung-RADS 3: Probably benign. Low risk of malignancy (cancer) 1%-2%.
  Radiologist: There is a 7-mm solid nodule in the left lung. Lung-RADS 3: Probably benign. Low risk of malignancy (cancer) 1%-2%.
- Recommendation
  Repeat low-dose CT in 6 months to ensure no interval growth, based on AI and radiologist agreement.

**Radiologist overcalls AI**

- Impression
  LungDetect AI: There is a 7-mm solid nodule in the left lung. Lung-RADS 3: Probably benign. Low risk of malignancy (cancer) 1%-2%.
  Radiologist: There is a 9-mm solid nodule in the left lung. Lung-RADS 4A: Suspicious. Medium risk of malignancy (cancer) 5%-15%.
- Recommendation
  LungDetect AI: Repeat low-dose CT in 6 months to ensure no interval growth.
  Radiologist: Undergo additional imaging immediately.

**Radiologist undercalls AI**

- Impression
  LungDetect AI: There is a 9-mm solid nodule in the left lung. Lung-RADS 4A: Suspicious. Medium risk of malignancy (cancer) 5%-15%.
  Radiologist: There is a 7-mm solid nodule in the left lung. Lung-RADS 3: Probably benign. Low risk of malignancy (cancer) 1%-2%.
- Recommendation
  LungDetect AI: Undergo additional imaging immediately.
  Radiologist: Repeat low-dose CT in 6 months to ensure no interval growth.

Participants chose their preferred next examination (repeat CT in 6 months or PET-CT now) and rated how much they agreed with the radiologist’s recommendation. They also rated their satisfaction with the radiologist. Participants then answered questions about their attitudes toward medical AI and their MMM preferences. Refer to Multimedia Appendix 1 for the hypothetical scenario and questionnaire measures.

The main dependent variable was a single, 6-point Likert scale measure asking participants how much they agreed or disagreed with the radiologist’s recommendation (1=strongly disagree to 6=strongly agree). We also measured participants’ satisfaction with their radiologist using questions adapted from the Agency for Healthcare Research and Quality’s Consumer Assessment of Healthcare Providers and Systems patient-experience surveys [26]. These included two 10-point scales asking participants how likely they would be to recommend the radiologist to family and friends and how good of a provider they perceived the radiologist.

Covariates included mean composite scores of participants’ attitudes toward medical AI and of participants’ MMM preferences. Attitudes toward medical AI were measured by asking participants to rate their agreement with 7 statements on a 6-point Likert scale (1=strongly disagree to 6=strongly agree). Of these statements, 5 were adapted from previous literature [27,28], and 2 were added by the authors. We reverse-coded statements 2 through 6 before creating the composite so that higher scores represented greater affinity for medical AI. Cronbach α for the 7 measures was 0.62, indicating acceptable internal consistency.

We measured MMM using 2 previously published questions [17,29]. The measures asked, in medical situations, whether participants: (1) tended to lean toward waiting and seeing or taking action (1=I strongly lean toward waiting and seeing to 6=I strongly lean toward taking action) [17], and (2) tended to lean toward doing only what is necessary or everything possible (1=I strongly lean toward doing only what is necessary to 6=I strongly lean toward doing everything possible) [29]. Higher scores on these measures indicated a greater preference for medical maximizing. Cronbach α for the 2 measures was 0.74, indicating good internal consistency. We also categorized participants as minimizers (1-2.5), neutrals (3-4), and maximizers (4.5-6), in line with previous research [29,30], for ease of interpretation.

### Analysis

We conducted ordinary least squares regressions to test whether receiving a recommendation from a radiologist alone, or in agreement or disagreement with AI, affected participants’ agreement with their radiologist’s recommendation. Two-sided *P*<.05 was considered significant. In exploratory analyses, we also tested how the experimental condition affected participants’ follow-up test choice and participants’ satisfaction with their radiologist.

In sensitivity analyses, we conducted Kruskal-Wallis H tests, Dunn tests, and ordinal logistic regression models to check the robustness of the effects on our main dependent variable, agreement with the radiologist’s recommendation. We also conducted regressions with the inclusion of medical AI attitudes, MMM preferences, and participant demographics as covariates to check the sensitivity of the findings to the inclusion of controls. We ran moderation analyses with participants’ MMM preferences first as a continuous variable and second as a categorical variable.

### Ethical Considerations

The study was declared exempt from review by the University of Michigan Health Sciences and Behavioral Sciences institutional review board.

## Results

### Descriptive Data

The average time to completion was 8 minutes 32 seconds after excluding 222 speeder participants who completed the questionnaire in 3 minutes or less. A total of 1606 (88%) participants (51% female, mean age 51.49 years) were used in the analyses. Participants in each condition were similar in mean age and sex, indicating successful randomization (Table 1).

**Table 1:** Descriptive statistics for the sample (N=1606).

Characteristic			Radiologist only (n=413)	Radiologist-AI^a^ agreement (n=401)	Radiologist overcalls AI (n=408)	Radiologist undercalls AI (n=384)	Total (N=1606)	*P* value^b^	
Age (years), mean			52.15	50.79	51.29	51.72	51.49	.69	
**Gender, n (%)**									.67
	Women		215 (52)	206 (51)	219 (54)	183 (48)	823 (51)		
	Men		194 (47)	192 (48)	187 (46)	199 (52)	772 (48)		
	Other		4 (1)	3 (1)	2 (0)	2 (1)	11 (1)		
**Highest level of completed education, n (%)**									.28
		High school or less	80 (19)	97 (24)	90 (22)	77 (20)	344 (21)		
		Some postsecondary	133 (32)	127 (32)	117 (29)	137 (36)	514 (32)		
		Bachelor’s	125 (30)	105 (26)	127 (31)	108 (28)	465 (29)		
		Graduate	73 (18)	67 (17)	74 (18)	60 (16)	274 (17)		
		Other	2 (0)	5 (1)	0 (0)	2 (1)	9 (1)		
**Race, n (%)**									.76
	White		314 (76)	286 (71)	301 (74)	290 (76)	1191 (74)		
	Black		41 (10)	55 (14)	50 (12)	50 (13)	196 (12)		
	Asian		22 (5)	22 (5)	24 (6)	17 (4)	85 (5)		
	Multiracial or other		36 (9)	38 (9)	33 (8)	27 (7)	134 (8)		
**Hispanic or Latino, n (%)**									.42
	Not Hispanic		332 (80)	314 (78)	344 (84)	317 (83)	1307 (81)		
	Hispanic		78 (19)	82 (20)	60 (15)	63 (16)	283 (18)		
	Other		3 (1)	5 (1)	4 (1)	4 (1)	16 (1)		

^a^AI: artificial intelligence.

^b^ANOVA for age, chi-square tests for gender, education, race, and Hispanic/Latino. Some percentages may total >100 due to rounding.

### Main Results

#### Participants’ Agreement With the Radiologist’s Recommendation

Participants were less likely to agree with the radiologist who undercalled AI (mean 4.01, SE 0.07), which was significantly different than the other conditions (*P*<.001). Participants’ agreement with their radiologist’s recommendation was not significantly different among the radiologist-only (mean 4.57, SE 0.06), radiologist-AI agreement (mean 4.55, SE 0.07), and radiologist-overcalls-AI (mean 4.63, SE 0.06) conditions. Results were robust to the inclusion of covariates, including medical AI attitudes, MMM preferences, and participant demographics (Table S1 in Multimedia Appendix 1), and remained consistent when using alternative model specifications, including the Kruskal-Wallis H tests and Dunn tests and ordinal logistic regressions (Tables S2 and S3 Multimedia Appendix 1).

#### Moderation by Medical Maximizing-Minimizing Preferences

**Figure 1:**
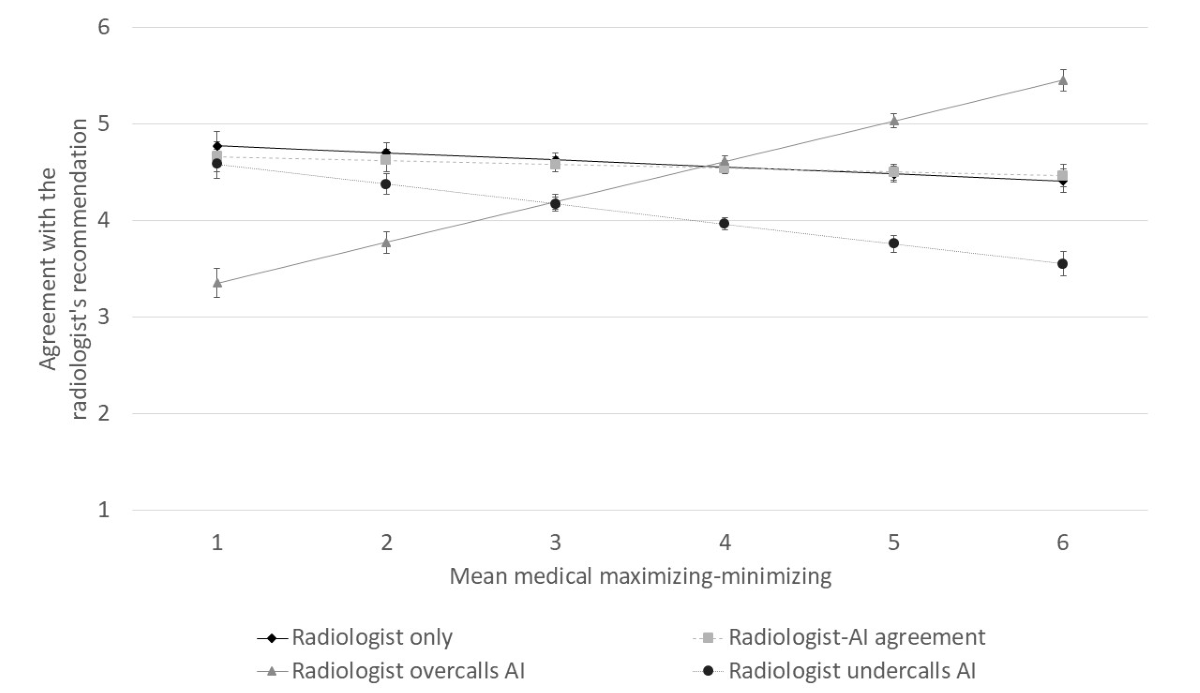
Effect of participants’ medical maximizing-minimizing preferences on agreement with the radiologist’s recommendation, by condition. Error bars depict SEs. AI: artificial intelligence.

MMM scores had a strong positive effect on participants’ agreement in the radiologist-overcalls-AI condition (β=0.42, SE 0.05, *P*<.001) and a negative effect on the radiologist-undercalls-AI condition (β=–0.21, SE 0.05, *P*<.001). There was no significant effect on the radiologist-only (β=–0.07, SE 0.05, *P*=.13) and radiologist-AI agreement (β=–0.04, SE 0.05, *P*=.42) conditions (Figure 1).

**Figure 2:**
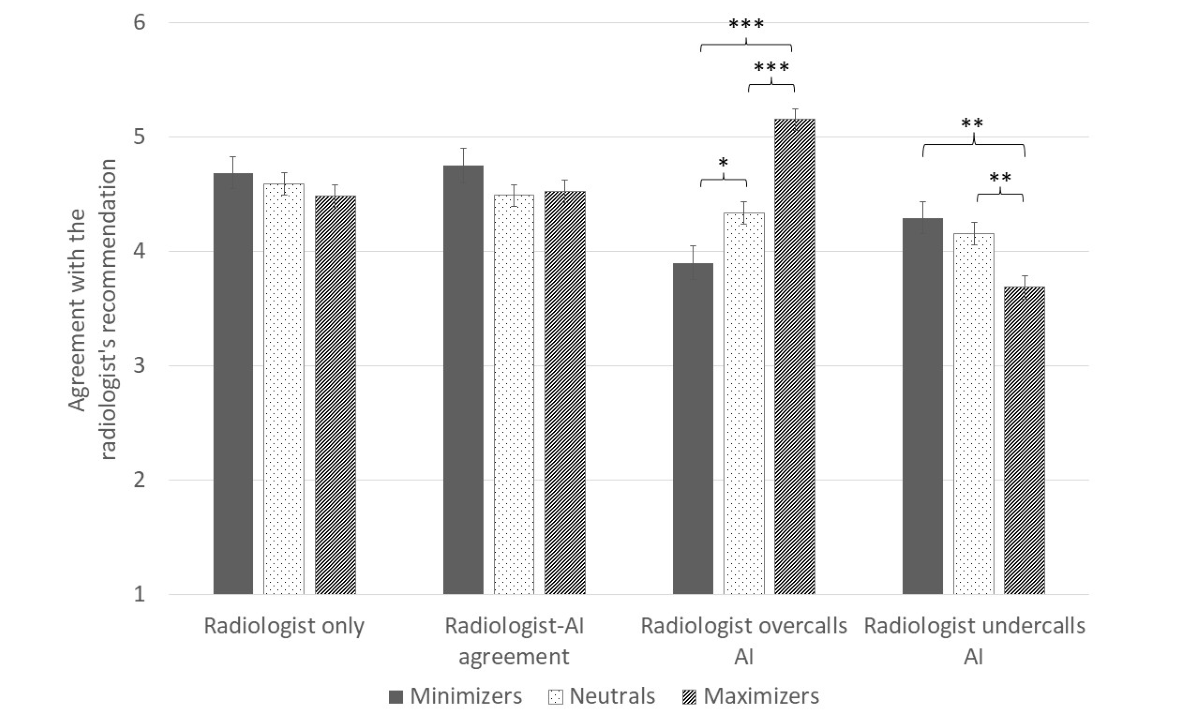
Effect of condition on participants’ agreement with the radiologist’s recommendation, by participants’ MMM category. Means and standard error bars are depicted. Stars represent significant differences between groups (*P<.05, **P<.01, ***P<.001). AI: artificial intelligence; MMM: medical maximizing-minimizing.

In our sample, MMM scores were left-skewed; 302 (18.8%) participants were minimizers, 654 (40.72%) were neutrals, and 650 (40.47%) were maximizers. When analyzing by MMM category, we found, relative to neutrals, minimizers disagreed more strongly with the radiologist who overcalled AI (β=–0.43, SE 0.18, *P*=.02) but did not agree more strongly with the radiologist who undercalled AI (β=0.14, SE 0.17, *P*=.41). Relative to neutrals, maximizers agreed more strongly with the radiologist who overcalled AI (β=0.82, SE 0.14, *P*<.001) and disagreed more strongly with the radiologist who undercalled AI (β=–0.47, SE 0.14, *P*=.001; Figure 2). The pattern of this moderation analysis with MMM as a categorical variable matched that of the analysis using MMM as a continuous variable.

### Secondary Results

#### Patients’ Choice Between Follow-Up Screening Computed Tomography in 6 Months or Additional Testing (PET-CT) Immediately

Participants were least likely to follow the recommendation of the radiologist who undercalled AI (184/384, 47.9%), which was significantly different than the other conditions (*P*<.001). Participants’ choice to follow their radiologist’s recommendation was not significantly different among the radiologist-only (260/413, 63%), radiologist-AI agreement (254/401, 63.3%), and radiologist-overcalls-AI conditions (263/408, 64.5%; Figure S1 in Multimedia Appendix 1). This pattern aligned with the results of participants’ agreement with their radiologist’s recommendation.

#### Patients’ Likelihood to Recommend the Radiologist and Radiologist Rating

In exploratory analyses, we examined participants’ likelihood to recommend their radiologist and the rating of their radiologist after receiving the results of their LDCT scan. Table 2 presents means and SEs for all dependent variables. Participants were both least likely to recommend (mean 6.47, SE 0.13) and positively rate (mean 6.82, SE 0.11) the radiologist who undercalled AI, which was significantly different from the other conditions (*P*<.001). There were no significant differences among the radiologist-only, radiologist-AI agreement, and radiologist-overcalls-AI conditions in participants’ recommendation likelihood (mean 7.24, SE 0.12 vs mean 7.40, SE 0.12 vs mean 7.43, SE 0.11) or rating of the radiologist (mean 7.45, SE 0.10 vs mean 7.55, SE 0.10 vs mean 7.58, *SE*=0.10). Results were robust to the inclusion of covariates, including AI attitudes, MMM preferences, and participant demographics. (Tables S4 and S5 in Multimedia Appendix 1).

**Table 2:** Means and standard errors of the dependent variables.

Dependent variable	Radiologist only (n=413)	Radiologist-AI^a^ agreement (n=401)	Radiologist overcalls AI (n=408)	Radiologist undercalls AI (n=384)
Agreement with radiologist’s recommendation^b^	4.57 (0.06)	4.55 (0.07)	4.63 (0.06)	4.01 (0.07)
Likelihood to recommend radiologist^b^	7.24 (0.12)	7.40 (0.12)	7.43 (0.11)	6.47 (0.13)
Rating of radiologist^b^	7.45 (0.10)	7.55 (0.10)	7.58 (0.10)	6.82 (0.11)

^a^AI: artificial intelligence.

**^b^**The radiologist-undercalls-AI group significantly differed from the three other groups on this dependent variable (*P*<.001). Agreement with the radiologist’s recommendation measured from 1=strongly disagree to 6=strongly agree. The likelihood to recommend the radiologist measured from 1=definitely would not recommend to 10=definitely would recommend. The rating of radiologist measured from 1=worst provider possible to 10=best provider possible.

#### Moderation by Medical Maximizing-Minimizing Preferences

MMM scores had a positive effect on participants’ likelihood to recommend the radiologist in the radiologist-overcalls-AI condition (β=0.71, SE 0.09, *P*<.001). Relative to neutrals, maximizers in the radiologist-overcalls-AI condition were more likely to recommend the radiologist (β=1.49, SE 0.26, *P*<.001).

MMM scores also had a positive effect on participants’ ratings of the radiologist in the radiologist-AI agreement (β=0.22, SE 0.08, *P*=.005) and the radiologist-overcalls-AI (β=0.61, SE 0.07, *P*<.001) conditions. Relative to neutrals, minimizers in the radiologist-overcalls-AI condition gave lower radiologist ratings (β=–0.63, SE 0.28, *P*=.03) whereas maximizers gave higher ratings (β=1.28, SE 0.22, *P*<.001). In addition, relative to neutrals, maximizers in the radiologist-undercalls-AI condition gave lower radiologist ratings (β=–0.49, SE 0.23, *P*=.03) (Figures S2 and S3 in Multimedia Appendix 1).

## Discussion

### Principal Findings

In this study, we found that participants penalized radiologists who undercalled AI in favor of less testing but not radiologists who overcalled AI in favor of more testing. Maximizers especially disagreed with radiologists who undercalled AI. However, whereas maximizers agreed with radiologists who overcalled AI, minimizers strongly disagreed. This suggests people’s confidence in the expertise of radiologists who use AI may depend in part on whether their general preferences to maximize or minimize align with the provided recommendations.

Previous research has shown that patients demonstrate algorithm aversion and superior trust in their human clinicians versus AI [8,31-33]. Our research suggests this may not always be the case. When AI and human clinicians are at odds, patients may trust AI over the clinician when AI detects something more than the clinician and recommends more aggressive treatment.

Although radiologists were penalized for undercalling AI, they were not penalized for overcalling AI. This overall attitude toward the radiologist who overcalled AI masked significant variability based on participants’ MMM preferences. Whereas minimizers penalized the radiologist, maximizers rewarded the radiologist. The averaging of these competing attitudes resulted in a level of agreement that did not significantly differ from that in the radiologist-only condition. Thus, radiologists who overcall AI may in fact face significantly lowered trust from patients who have general minimizing preferences.

We did not observe a similar aggregate averaging effect in the radiologist-undercalls-AI condition. This asymmetry may be explained by the perceptions of minimizers. Whereas maximizers demonstrated stable preferences for pursuing more aggressive treatment [30], regardless of whether the radiologist or AI recommended it, minimizers penalized the radiologist who overcalled AI but did not reward the radiologist who undercalled AI.

Minimizers typically prefer not to receive treatment unless considered essential [30,34]. However, previous research has shown minimizers are more responsive to information than maximizers and will change their attitudes in response to evidence [18]. Thus, 1 theory for this asymmetry is that minimizers perceived AI as more accurate than the radiologist and therefore deferred to the AI’s recommendation both when AI recommended more and less testing. This suggests patients, like clinicians, may be prone to automation bias when medical AI is involved [35,36]. Another theory is that the emotional salience of the AI’s cancer results softened minimizers’ attitudes toward more testing. Importantly, patients demonstrated higher agreement in the radiologist-only and radiologist-AI agreement conditions, despite the radiologist recommending less testing just like in the radiologist-undercalls-AI condition. This suggests participants were opposed to the radiologist’s recommendation only when in contrast with the more aggressive AI recommendation. Research suggests the emotional salience of a cancer diagnosis, particularly fear of cancer, influences patients’ decision-making [37-39]. More research is needed to test these theories, including examining patients’ general and context-specific perceptions of the accuracy of AI compared with that of clinicians [31].

Radiologists’ confirmatory “second opinion” of AI did not boost participants’ agreement with them. This finding adds a new dimension to the literature examining patient perceptions of human- versus model-assisted decisions. In 1 study, patients’ trust in AI increased when AI, acting as a second opinion, confirmed the human clinician’s diagnosis, but decreased when the AI was disconfirming [32]. In another study, participants devalued clinicians who consulted a computer-based diagnostic aid for a second opinion, but not clinicians who consulted a human expert [40]. In this study, we found radiologists who agreed with the AI were neither rewarded nor penalized compared with the radiologist alone.

Research shows the human-AI team augments the diagnostic performance of the radiologist [41,42]. However, we show that the human-AI team may not benefit, and could even hurt, the radiologist in terms of patient agreement or satisfaction. This study demonstrated that even small discrepancies (ie, 2 mm) between AI and radiologist interpretations can elicit strong patient reactions. We anticipate larger discrepancies would result in more pronounced negative reactions toward clinicians. As a result, clinicians may feel compelled to overcall or agree with AI to avoid negative patient reactions, as well as potential legal consequences [43,44]. This may lead to incorrect clinician decisions [45], as well as unnecessary testing, biopsies, and procedures. More research is needed to probe clinicians’ attitudes toward the consequences of using AI.

Future research is also needed to better understand patient attitudes toward the human-AI team in various clinical contexts. Research should also interrogate what and how to communicate AI results with patients, especially when the human-AI team disagrees. Offering 2 recommendations to patients may eschew clinicians’ professional agency and burden patients with a choice between following clinician or AI expertise [46]. However, there are ethical considerations to smoothing over discrepancies with a single recommendation that sides with either the clinician or the AI. If both recommendations are medically reasonable, a single clinician recommendation could be seen as too paternalistic [47] and may go against patient preference [48].

### Limitations

This study has several limitations. First, we intentionally recruited a diverse sample of US adults to receive the broadest range of participants; however, this sample is not reflective of the patients who typically would receive LDCT scans. Future research should consider replicating this study with a narrowed sample of participants, particularly smokers, or real patients undergoing cancer screening. For example, we anticipate participants with a higher pretest probability for lung cancer would find a radiologist undercalling AI particularly difficult to accept in this scenario.

Second, we instructed participants that they were eligible to receive an LDCT scan because of “personal risk factors” but did not specify further. Risk factors could range from genetic to environmental to behavioral, and how we develop these risk factors influences our acceptance of test results [49,50]. If participants had different mental models for how they would be eligible for lung cancer screening, these differences could have impacted their responses to the AI and radiologist’s recommendations.

Third, although studies show patients want to know and consent to when AI is used [27,51], we do not yet have a template for how health care systems currently report, will report in the future, or should report AI use to patients. In our study, the EHR results and oral follow-up provided both the AI and radiologist’s interpretations and recommendations. A different presentation of AI’s involvement, such as AI’s interpretation but not a separate recommendation, or simply that AI was consulted but not its interpretation, could elicit different patient perceptions of their radiologist. We also do not provide measures of accuracy for either AI or radiologists. Although radiologists’ accuracy is not typically disclosed to patients who receive imaging, it is possible that patients may inquire about the AI’s accuracy, especially vis-à-vis the radiologists'. How patients understand and interpret measures of accuracy, including more advanced measures, such as sensitivity, specificity, precision, and recall, requires further investigation.

Furthermore, future research is needed to develop validated instruments for measuring attitudes toward medical AI. Although there are validated scales measuring attitudes toward AI in general [52,53], these were insufficient for our experimental context. Thus, the scale we deployed, for which we created a composite score from custom-designed questions and achieved a low Cronbach α, was neither validated nor highly reliable, a limitation of our study. As a result, we may not have adequately controlled for confounding medical AI attitudes, weakening our overall findings.

Finally, the radiologist-patient relationship differs from the classic clinician-patient relationship, as patients might not choose, directly communicate with, or have a preexisting relationship with their radiologist [54,55]. The nature of the radiologist-patient relationship may therefore not generalize to other medical specialties. Despite these limitations, exploring patients’ attitudes toward cancer screening recommendations and the radiologist-patient relationship are understudied yet increasingly important topics in the era of medical AI [56].

### Conclusion

As radiologists begin to integrate AI into cancer detection, discrepancies within the human-AI team may influence patients’ reactions toward their radiologist. People may penalize radiologists who undercall AI in lung cancer screening yet may not be more confident in radiologists who overcall or agree with AI. Patients’ MMM preferences moderate this effect and accounting for patients’ MMM preferences may give insight to clinicians into what and how much information to give about AI’s role in their decision-making. Our findings highlight the complexity of the patient-AI-clinician relationship and have implications for clinical practice, communication, and shared decision-making. Future research is needed to determine how radiologists should communicate AI discrepancies to patients in a way that builds trust and maintains their relevance.

## Appendix

Multimedia Appendix 1 Additional material.

## Abbreviations

AI: artificial intelligence
CT: computerized tomography
EHR: electronic health record
FDA: US Food and Drug Administration
HITL: human in the loop
LDCT: low-dose computerized tomography
MMM: medical maximizing-minimizing
PET: positron emission tomography

## Data Availability

The dataset generated for this study is available from the corresponding author on request.
