# Supplementary material for: Patient Reactions to Artificial Intelligence–Clinician Discrepancies: Web-Based Randomized Experiment
Source: J Med Internet Res. 2025 May 22;27:e68823. doi: 10.2196/68823 (PMC12141964; doi:10.2196/68823)
Supplement: Multimedia Appendix 1 [file jmir_v27i1e68823_app1.docx]

**Supplementary Material**

Supplementary Table 1: Agreement with the radiologist’s recommendation, by condition, with added covariates, using OLS models

|  | Model 1 | Model 2 | Model 3 | Model 4 | Model 5 | Model 6 |
| --- | --- | --- | --- | --- | --- | --- |
| Condition  Radiologist only  Radiologist-AI agreement Radiologist overcall AI Radiologist undercalls AI | ref -0.02 (0.09)  0.05 (0.09) -0.56 (0.09)*** | ref -0.04 (0.09) 0.06 (0.09) -0.53 (0.09)*** | ref -0.03 (0.09) 0.05 (0.09) -0.56 (0.09)*** | ref -0.008 (0.09) 0.05 (0.09) -0.53 (0.09)*** | ref -0.009 (0.09) 0.06 (0.09) -0.58 (0.09)*** | ref 0.009 (0.09) 0.06 (0.09) -0.55 (0.09)*** |
| Medical AI attitudes |  | -0.19 (0.04)*** |  | -0.18 (0.04)*** |  | -0.19 (0.04)*** |
| MMM |  |  | 0.04 (0.02) | 0.04 (0.02) |  | 0.03 (0.02) |
| Age |  |  |  |  | -0.0014 (0.002) | -0.001 (0.002) |
| Gender  Women Men Other |  |  |  |  | ref 0.07 (0.06) -0.38 (0.41) | ref 0.100 (0.06) -0.29 (0.41) |
| Education  HS or less Some postsecondary Bachelor’s Graduate Other |  |  |  |  | ref 0.01 (0.09) 0.11 (0.09) 0.04 (0.11) -0.14 (0.46) | ref 0.02 (0.09) 0.13 (0.09) 0.07 (0.11) -0.13 (0.45) |
| Race  White Black Asian Other |  |  |  |  | ref 0.02 (0.10) -0.18 (0.14) -0.41 (0.13)** | ref 0.03 (0.10) -0.16 (0.14) -0.41 (0.13)** |
| Hispanic/Latino  Not Hispanic Hispanic  Other |  |  |  |  | ref 0.04 (0.09) -0.78 (0.35)* | ref 0.06 (0.09) -0.71 (0.34)* |
| Constant | 4.57 (0.06)*** | 5.14 (0.15)*** | 4.41 (0.11)*** | 5.00 (0.18)*** | 4.60 (0.14)*** | 5.04 (0.21)*** |
| Notes: MMM = Medical maximizing-minimizing **P*<.05, ***P*<.01, ****P*<.001 | | | | | | |

**Sensitivity Analyses**

**Kruskal-Wallis H Test and Dunn’s Test**

A Kruskal-Wallis H test showed a significant effect of experimental condition on participants’ agreement with the radiologist’s recommendation, χ²(3) = 51.97, *P*<.001. Post-hoc Dunn’s pairwise comparisons with Bonferroni correction revealed that participants in the radiologist-undercalls-AI condition had significantly different agreement with the radiologist’s recommendation than participants did in the other conditions (*P*<.001), whereas the other comparisons were not statistically significant (*P*>.05).

Supplementary Table 2: Dunn’s test results for agreement with the radiologist’s recommendation, by condition

| **Condition comparison** | **Z (Dunn’s Test)** | **p-value (Bonferroni-corrected)** |
| --- | --- | --- |
| Radiologist only vs. Radiologist-AI agreement | -0.33 | 1.00 |
| Radiologist only vs. Radiologist overcalls AI | -0.43 | 1.00 |
| Radiologist-AI agreement vs. Radiologist overcalls AI | -.09 | 1.00 |
| Radiologist only vs. Radiologist undercalls AI | 5.89 | <.001 |
| Radiologist-AI agreement vs. Radiologist undercalls AI | 6.18 | <.001 |
| Radiologist overcalls AI vs. Radiologist undercalls AI | 6.30 | <.001 |

Supplementary Table 3: Agreement with the radiologist’s recommendation, by condition, with added covariates, using ordinal logitic regression

|  | Model 1 OR (95% CI) | Model 2 OR (95% CI) | Model 3 OR (95% CI) | Model 4 OR (95% CI) | Model 5 OR (95% CI) | Model 6 OR (95% CI) |
| --- | --- | --- | --- | --- | --- | --- |
| Condition  Radiologist only  Radiologist-AI agreement Radiologist overcall AI Radiologist undercalls AI | ref 1.05 (0.82–1.34) 1.06 (0.83–1.35) 0.47 (0.36–0.60)*** | ref 1.09 (0.85–1.39) 1.08 (0.84–1.38) 0.49 (0.38–0.63)*** | ref 1.04 (0.81–1.33) 1.03 (0.80–1.31) 0.47 (0.36–0.60)*** | ref 1.08 (0.84–1.39) 1.05 (0.82–1.34) 0.49 (0.38–0.63)*** | ref 1.06 (0.83–1.36) 1.07 (0.83–1.37) 0.46 (0.36–0.59)*** | ref 1.10 (0.86–1.41) 1.07 (0.83–1.37) 0.49 (0.38–0.63)*** |
| Medical AI attitudes |  | 0.72 (0.64–0.82)*** |  | 0.73 (0.64–0.83)*** |  | 0.72 (0.63–0.82)*** |
| MMM |  |  | 1.12** | 1.11 (1.04–1.20)** |  | 1.10 (1.03–1.19)** |
| Age |  |  |  |  | 1.00 (0.99–1.00) | 1.00 (0.99–1.01) |
| Gender  Women Men Other |  |  |  |  | ref 1.09 (0.91–1.30) 0.56 (0.18–1.78) | ref 1.15 (0.96–1.37) 0.70 (0.22–2.19) |
| Education  HS or less Some postsecondary Bachelor’s Graduate Other |  |  |  |  | ref 0.95 (0.74–1.23) 1.10 (0.85–1.42) 1.04 (0.78–1.40) 0.96 (0.21–4.38) | ref 0.96 (0.74–1.23) 1.12 (0.87–1.45) 1.07 (0.80–1.45) 0.95 (0.20–4.47) |
| Race  White Black Asian Other |  |  |  |  | ref 1.07 (0.80–1.43) 0.76 (0.51–1.12) 0.62 (0.43–0.88)** | ref 1.06 (0.79–1.42) 0.78 (0.53–1.15) 0.61 (0.43–0.88)** |
| Hispanic/Latino  Not Hispanic Hispanic  Other |  |  |  |  | ref 1.00 (0.78–1.29) 0.32 (0.12–0.80)* | ref 1.03 (0.80–1.33) 0.37 (0.15–0.94)* |
| Notes: MMM = Medical maximizing-minimizing **P*<.05, ***P*<.01, ****P*<.001 | | | | | | |

Supplementary Table 4: Likelihood to recommend the radiologist, by condition, with added covariates, using OLS models

|  | Model 1 | Model 2 | Model 3 | Model 4 | Model 5 | Model 6 |
| --- | --- | --- | --- | --- | --- | --- |
| Condition  Radiologist only  Radiologist-AI agreement Radiologist overcall AI Radiologist undercalls AI | ref 0.16 (0.17) 0.19 (0.17) -0.76 (0.17)*** | ref 0.18 (0.17) 0.20 (0.17) -0.73 (0.17)*** | ref 0.14 (0.17) 0.16 (0.17) -0.76 (0.17)*** | ref 0.16 (0.17) 0.17 (0.17) -0.73 (0.17)*** | ref 0.19 (0.17) 0.23 (0.17) -0.76 (0.17)*** | ref 0.19 (0.17) 0.21 (0.17) -0.72 (0.17)*** |
| Medical AI attitudes |  | -0.23 (0.08)** |  | -0.21 (0.08)** |  | -0.21 (0.08)* |
| MMM |  |  | 0.20 (0.05)*** | 0.20 (0.05)*** |  | 0.19 (0.05)*** |
| Age |  |  |  |  | -0.01 (0.00) | -0.01 (0.00) |
| Gender  Women Men Other |  |  |  |  | ref 0.01 (0.12) -0.90 (0.78) | ref 0.06 (0.12) -0.73 (0.77) |
| Education  HS or less Some postsecondary Bachelor’s Graduate Other |  |  |  |  | ref 0.30 (0.17) 0.25 (0.17) 0.20 (0.20) 0.19 (0.86) | ref 0.30 (0.17) 0.25 (0.17) 0.18 (0.20) 0.19 (0.85) |
| Race  White Black Asian Other |  |  |  |  | ref -0.38 (0.19)* -0.11 (0.27) -0.60 (0.24)* | ref -0.41 (0.19)* -0.10 (0.27) -0.61 (0.24)* |
| Hispanic/Latino  Not Hispanic Hispanic  Other |  |  |  |  | ref 0.28 (0.17) -1.60 (0.65)* | ref 0.32 (0.17) -1.39 (0.65)* |
| Constant | 7.24 (0.12)*** | 7.95 (0.27)*** | 6.46 (0.21)*** | 7.14 (0.33)*** | 7.41 (0.26)*** | 7.27 (0.39)*** |
| Notes: MMM = Medical maximizing-minimizing **P*<.05, ***P*<.01, ****P*<.001 | | | | | | |

Supplementary Table 5: Rating of the radiologist, by condition, with added covariates, using OLS models

|  | Model 1 | Model 2 | Model 3 | Model 4 | Model 5 | Model 6 |
| --- | --- | --- | --- | --- | --- | --- |
| Condition  Radiologist only  Radiologist-AI agreement Radiologist overcall AI Radiologist undercalls AI | ref 0.10 (0.14) 0.13 (0.14) -0.63 (0.14)*** | ref 0.12 (0.14) 0.14 (0.14) -0.61 (0.14)*** | ref 0.09 (0.14) 0.10 (0.14) -0.63 (0.14)*** | ref 0.10 (0.14) 0.11 (0.14) -0.60 (0.14)*** | ref 0.12 (0.14) 0.16 (0.14) -0.63 (0.14)*** | ref 0.12 (0.14) 0.14 (0.14) -0.60 (0.14)*** |
| Medical AI attitudes |  | -0.16 (0.07)* |  | -0.14 (0.07)* |  | -0.13 (0.07) |
| MMM |  |  | 0.17 (0.04)*** | 0.17 (0.04)*** |  | 0.16 (0.04)*** |
| Age |  |  |  |  | 0.00 (0.00) | 0.00 (0.00) |
| Gender  Women Men Other |  |  |  |  | ref 0.01 (0.10) -1.29 (0.66) | ref 0.04 (0.10) -1.17 (0.66) |
| Education  HS or less Some postsecondary Bachelor’s Graduate Other |  |  |  |  | ref 0.14 (0.14) 0.10 (0.15) 0.06 (0.17) 0.64 (0.73) | ref 0.14 (0.14) 0.10 (0.15) 0.04 (0.17) 0.64 (0.72) |
| Race  White Black Asian Other |  |  |  |  | ref -0.13 (0.16) -0.22 (0.23) -0.61 (0.20)** | ref -0.16 (0.16) -0.21 (0.23) -0.62 (0.20)** |
| Hispanic/Latino  Hispanic Not Hispanic  Other |  |  |  |  | ref 0.28 (0.15) -1.25 (0.55)* | ref 0.32 (0.14)* -1.09 (0.55)* |
| Constant | 7.45 (0.10)*** | 7.93 (0.23)*** | 6.79 (0.18)*** | 7.24 (0.28)*** | 7.38 (0.22)*** | 7.12 (0.34)*** |
| Notes: MMM = Medical maximizing-minimizing **P*<.05, ***P*<.01, ****P*<.001 | | | | | | |


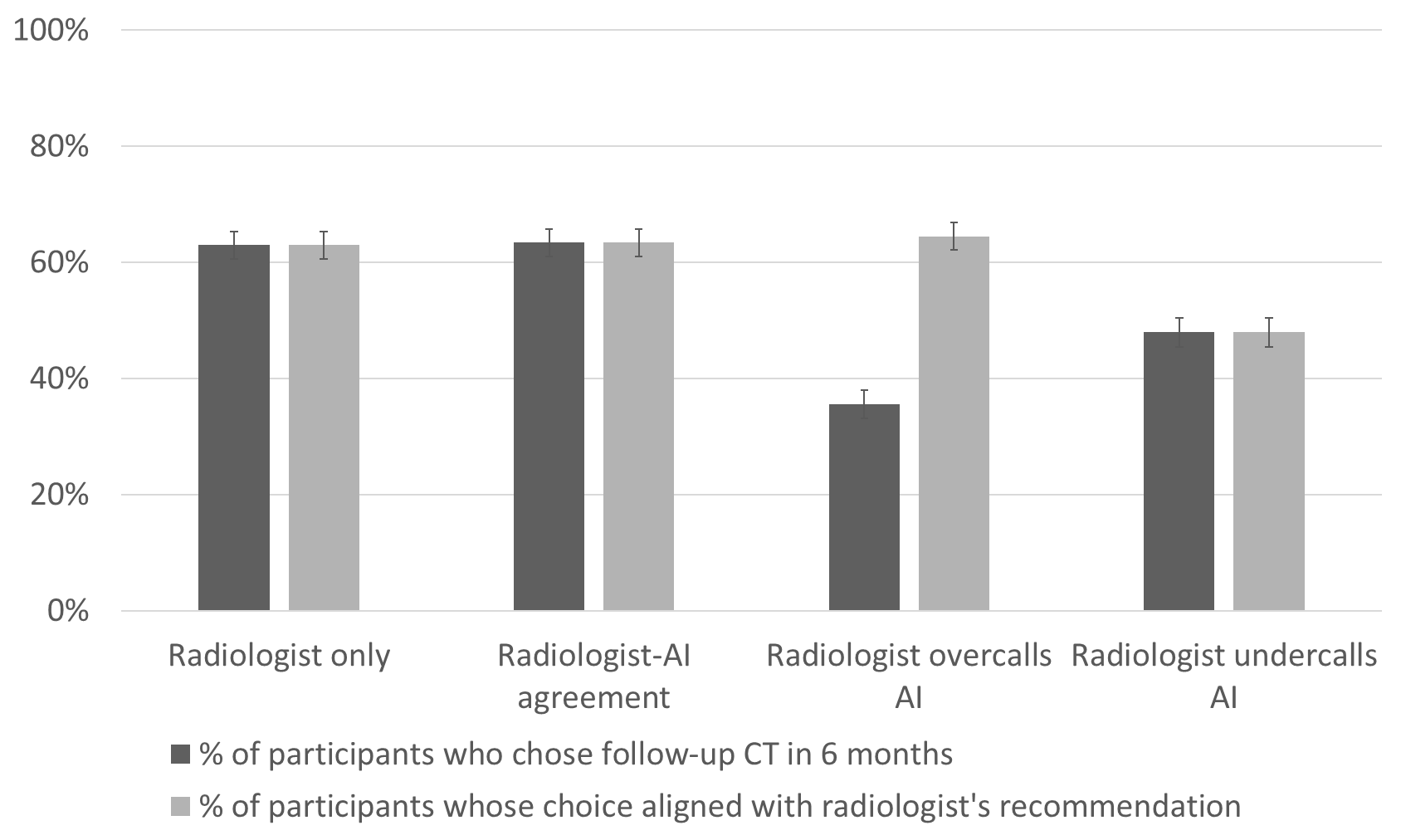


Supplementary Figure 1: Percentage of participants (1) who chose to receive the follow-up screening CT in 6 months (instead of immediate testing [PET/CT]) and (2) whose choice aligned with the radiologist’s recommendation, by condition. Percentages and standard error bars depicted.


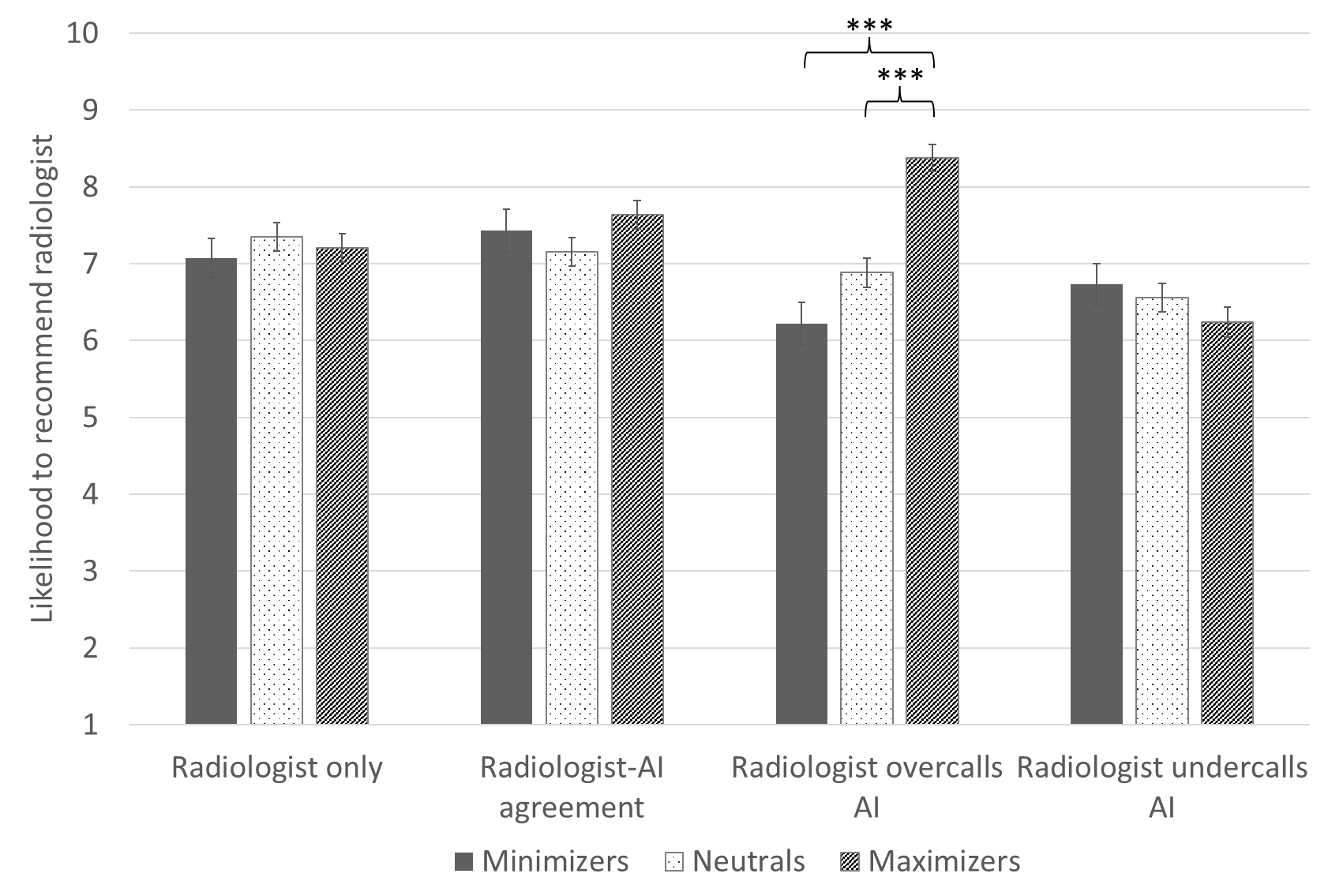


Supplementary Figure 2: Effect of condition on participants’ likelihood to recommend the radiologist, by MMM category. Means and standard error bars depicted. Stars represent significant differences between groups
(**P*<.05, ***P*<.01, ****P*<.001).

**
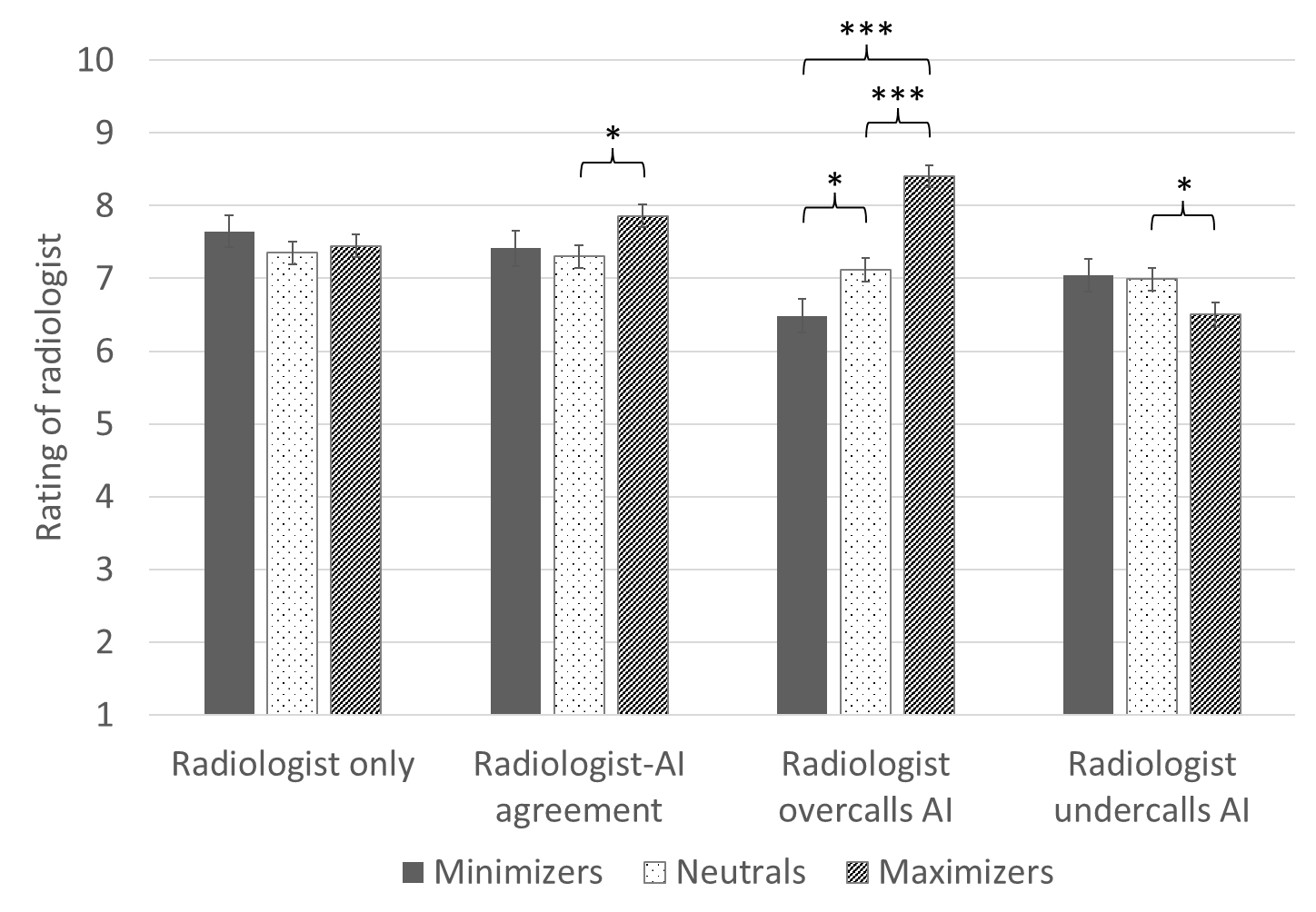
**Supplementary Figure 3: Effect of condition on participants’ rating of the radiologist, by MMM category. Means and standard error bars depicted. Stars represent significant differences between groups
(**P*<.05, ***P*<.01, ****P*<.001).

**Questionnaire Measures**

*Patients’ agreement with the radiologist’s recommendation:*

How much do you agree or disagree with **Dr. Smith’s recommendation to repeat the screening CT in 6 months [to undergo additional testing (PET/CT) immediately]**?

| 1  Strongly Disagree | 2  Disagree | 3  Slightly Disagree | 4  Slightly Agree | 5  Agree | 6  Strongly Agree |
| --- | --- | --- | --- | --- | --- |

*Patients’ satisfaction in the radiologist:*

How likely would you be to recommend your reading radiologist, **Dr. Smith**, to a friend or a family member (1 = definitely would not recommend, 10 = definitely would recommend)

| 1  Definitely would not recommend | 2 | 3 | 4 | 5 | 6 | 7 | 8 | 9 | 10  Definitely would recommend |
| --- | --- | --- | --- | --- | --- | --- | --- | --- | --- |

Using any number from 1 to 10, where 1 is the worst provider possible and 10 is the best provider possible, what number would you use to rate **Dr. Smith**?

| 1  Worst provider possible | 2 | 3 | 4 | 5 | 6 | 7 | 8 | 9 | 10  Best provider possible |
| --- | --- | --- | --- | --- | --- | --- | --- | --- | --- |

*Patients’ attitudes toward AI:*

Applications of AI in healthcare have increased in the past decade. Common applications include diagnosis, drug discovery, patient care, and remote treatment.

**How much do you agree or disagree with the following statements:**

1. Overall, in the next 5 years, AI will make health care better in the United States.^a^
2. I would trust the assessment of AI less than the assessment of a physician.^b^
3. A physician should always have final control over diagnoses and therapy.^b^
4. I am more afraid of a technical malfunction of AI than of a wrong decision by a physician.^b^
5. I would like my physician to override the recommendations of AI if they come to a different conclusion based on their experience or knowledge.^b^
6. I would prefer my physician not use AI.^c^
7. I want my physician to use all tools available to them, including AI.^c^

| 1  Strongly Disagree | 2  Disagree | 3  Slightly Disagree | 4  Slightly Agree | 5  Agree | 6  Strongly Agree |
| --- | --- | --- | --- | --- | --- |

*Notes:*

^a^Item adapted from: Khullar D, Casalino LP, Qian Y, Lu Y, Krumholz HM, Aneja S. Perspectives of patients about artificial intelligence in health care. *JAMA network open*. 2022;5(5):e2210309-e2210309.

^b^Fritsch SJ, Blankenheim A, Wahl A, et al. Attitudes and perception of artificial intelligence in healthcare: A cross-sectional survey among patients. *Digital health*. 2022;8:20552076221116772.

^c^Measures added by authors

*Patients’ medical maximizing-minimizing preferences:*

**MM1: Thinking about how much healthcare you prefer to get: What type are you?**

Sometimes, medical action is clearly necessary, and sometimes it is clearly NOT necessary. Other times, reasonable people differ in their beliefs about whether medical action is needed.

In situations where it’s not clear, do you tend to lean toward taking action or do you lean towards waiting and seeing if action is needed?

**Importantly, there is no “right” way to be.**

Please answer on the 1-6 scale below:

| 1  I strongly lean toward waiting and seeing | 2  I lean toward waiting and seeing | 3  I somewhat lean toward waiting and seeing | 4  I somewhat lean toward taking action | 5  I lean toward taking action | 6  I strongly lean toward taking action |
| --- | --- | --- | --- | --- | --- |

Scherer, L. D., & Zikmund-Fisher, B. J. (2020). Eliciting medical maximizing-minimizing preferences with a single question: development and validation of the MM1. *Medical Decision Making*, *40*(4), 545-550.

**MM2: When making decisions about medical care, do you tend to lean towards doing only what is necessary or do you lean towards doing everything possible?**

| 1  I strongly lean toward doing only what is necessary | 2  I lean toward doing only what is necessary | 3  I somewhat lean toward doing only what is necessary | 4  I somewhat lean toward doing everything possible | 5  I lean toward doing everything possible | 6  I strongly lean toward doing everything possible |
| --- | --- | --- | --- | --- | --- |

Dossett LA, Mott NM, Bredbeck BC, et al. Using tailored messages to target overuse of low-value breast cancer care in older women. *Journal of Surgical Research*. 2022;270:503-512.

**Questionnaire Hypothetical Scenario**

*Screening scenario presented to participants in each condition*

**Background - screening:**

You visit your primary care physician (PCP) for your annual wellness checkup. After the nurse does your intake and takes your vitals, your PCP comes in.

After some small talk your PCP says the following:

*Now, because of personal risk factors, you are at increased risk of lung cancer.* ***The guidelines recommend that you receive a low-dose screening CT scan.*** *So, I’m going to write you a referral for that CT. Is that something you’d be willing to do?*

You nod your head and ask what the scan entails. Your PCP explains:

*So, a CT, or computed tomography scan, is* ***indicated for lung cancer screening.*** *For the test, you will lie flat on your back on the CT scan table and remain still for about 20 seconds while the scanner moves quickly around your body taking X-ray images of your chest.* ***“Low dose” means it requires less radiation than a usual CT to see what we need.***

**Respondents are randomized to 1 of 4 conditions:**

1. Radiologist only
2. Radiologist and AI agreement
3. Radiologist overcalls AI
4. Radiologist undercalls AI

**Background – possible recommendations:**

*Presented to participants in the radiologist-only condition*

Your PCP continues:

*Your screening lung CT scan is then read by a radiologist.* ***The radiologist will interpret your scan and give a standardized recommendation*** *based on their concern for lung cancer. The radiologist may make one of four possible recommendations:*

1. *If the scan looks highly concerning,* ***you may be recommended a biopsy to be done as soon as possible.*** *A biopsy is a procedure in which tissue samples are removed to confirm whether a nodule, or a lump of cells, is cancer.*
2. *If the scan is suspicious,* ***you may be recommended additional imaging to be done as soon as possible.*** *Another imaging test, a PET/CT, would help us see how suspicious a nodule is.* ***However, there are downsides to additional imaging, so we don’t want to do this unless we must.*** *For example, a PET/CT, exposes you to a much higher level of radiation, and radiation is known to increase your risk of cancer. The test requires a special type of IV contrast, which is radioactive and can have significant side effects, such as pain, fever, nausea, and vomiting. You will also need to fast for 6 hours prior and drink an oral contrast when you arrive for your appointment.*
3. *If the scan shows a benign nodule or is too difficult to read,* ***you may be recommended a follow-up test, meaning another low-dose screening CT test, to be done in 6 months.***
4. *Last, if the scan is completely clear,* ***you may be recommended another low-dose screening CT test to be done in 1 year.***

*Presented to participants in the radiologist & AI conditions*

Your PCP continues:

*In our hospital, your screening lung CT scan is then read twice.*

*First, your screening lung CT scan is run through an* ***AI program,*** *called LungDetect AI, which is trained on thousands of CT images of lungs, both with and without cancer, to detect cancer in low-dose screening lung CT scans.*

*Then a* ***radiologist*** *will read and interpret the same scan.*

***Both the AI program and the radiologist will*** ***give a standardized recommendation*** *based on their concern for lung cancer. They each may make one of four possible recommendations:*

1. *If the scan looks highly concerning,* ***you may be recommended a biopsy to be done as soon as possible.*** *A biopsy is a procedure in which tissue samples are removed to confirm whether a nodule, or a lump of cells, is cancer.*
2. *If the scan is suspicious,* ***you may be recommended additional imaging to be done as soon as possible.*** *Another imaging test, a PET/CT, would help us see how suspicious a nodule is.* ***However, there are downsides to additional imaging, so we don’t want to do this unless we must.*** *For example, a PET/CT, exposes you to a much higher level of radiation, and radiation is known to increase your risk of cancer. The test requires a special type of IV contrast, which is radioactive and can have significant side effects, such as pain, fever, nausea, and vomiting. You will also need to fast for 6 hours prior and drink an oral contrast when you arrive for your appointment.*
3. *If the scan shows a benign nodule or is too difficult to read,* ***you may be recommended a follow-up test, meaning another low-dose screening CT test, to be done in 6 months.***
4. *Last, if the scan is completely clear,* ***you may be recommended another low-dose screening CT test to be done in 1 year.***

*Preamble to CT scan results presented to participants in each condition*

**Background – CT scan results:**

A week later, you go to your CT scan appointment. After the scan, you check out and are told you and your PCP will receive your results in a few days.

A few days later, you see an update in your medical chart online. You read the radiology report as follows:

**
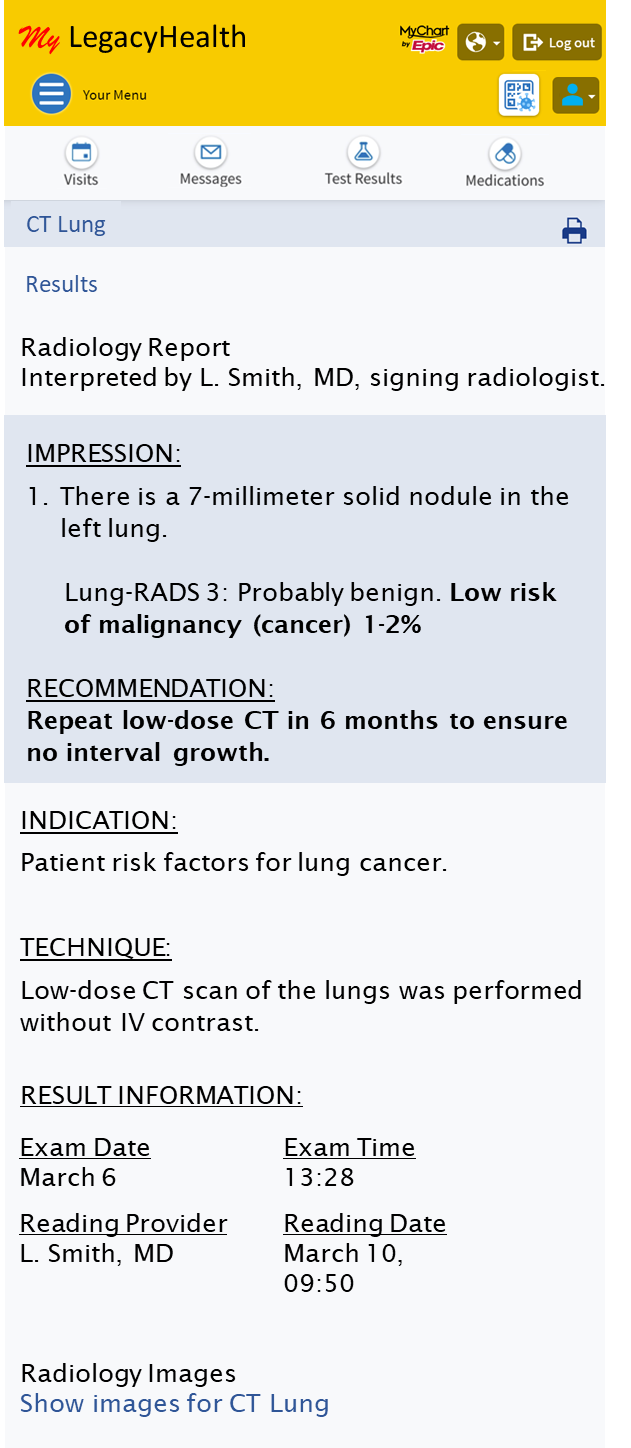
***Presented to participants in the radiologist-only condition*

Later that day, your PCP calls you to follow-up. He says:

*I wanted to call to follow-up. I saw you had your screening lung CT scan done last Wednesday.*

***Your reading radiologist, Dr. Smith, identified a nodule in your left lung that he believes is low risk for cancer based on its size, with only a 1-2% risk of being cancer.*** *Nodules like this are common on people’s first CT and may remain stable over time and never pose a risk of cancer.*

***Dr. Smith’s recommendation is to repeat the screening CT in 6 months.*** *This is to ensure the nodule is not growing. If the nodule remains stable, this will give us confidence that it is not cancer. If it does grow, we would recommend additional tests.*

**
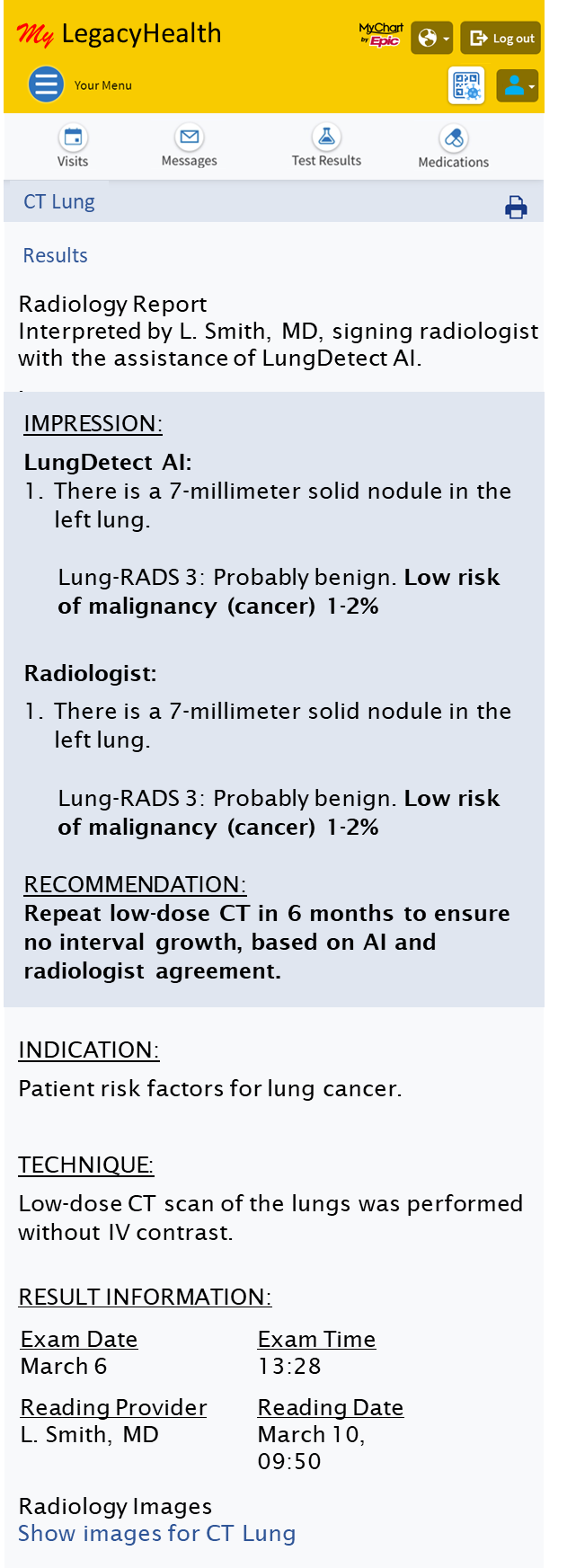
***Presented to participants in the radiologist-AI-agreement condition*

Later that day, your PCP calls you to follow-up. He says:

*I wanted to call to follow-up. I saw you had your screening lung CT scan done last Wednesday and* ***the AI and radiologist are in agreement****.*

***Our LungDetect AI program identified a nodule with a low risk of being cancer in your left lung.*** *The AI program is trained on thousands of low-dose CT images of lungs, both with and without cancer. Basically, the patterns on your scan match those in scans where the* ***patient ended up having cancer 1-2% of the time****.*

***LungDetect AI’s*** ***recommendation is to repeat the screening CT in 6 months****. This is to ensure the nodule is not growing. If the nodule remains stable, this will give us confidence that it is not cancer. If it does grow, we would recommend additional tests.*

***Your reading radiologist, Dr. Smith, identified the same nodule in your left lung, which he believes is low risk for cancer based on its size, with only a 1-2% risk of being cancer.*** *Nodules like this are common on people’s first CT and may remain stable over time and never pose a risk of cancer.*

*In agreement with LungDetect AI,* ***Dr. Smith’s recommendation is to repeat the screening CT in 6 months*** *to ensure no interval growth.*

**
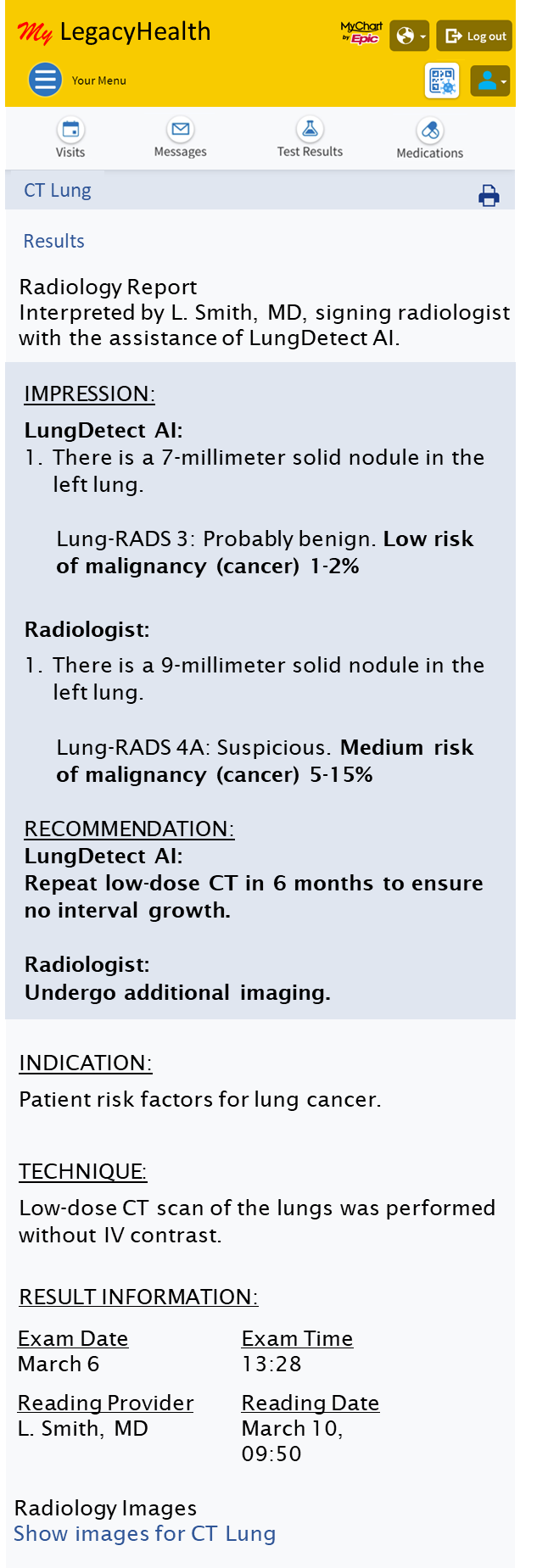
***Presented to participants in the radiologist-overcalls-AI condition*

Later that day, your PCP calls you to follow-up. He says:

*I wanted to call to follow-up. I saw you had your screening lung CT scan done last Wednesday and* ***the AI and radiologist are in disagreement****.*

***Our LungDetect AI program identified a nodule with a low risk of being cancer in your left lung.*** *The AI program is trained on thousands of low-dose CT images of lungs, both with and without cancer. Basically, the patterns on your scan match those in scans where the* ***patient ended up having cancer 1-2% of the time****.*

***LungDetect AI’s*** ***recommendation is to repeat the screening CT in 6 months****. This is to ensure the nodule is not growing. If the nodule remains stable, this will give us confidence that it is not cancer. If it does grow, we would recommend additional tests.*

***Your reading radiologist, Dr. Smith, identified the same nodule in your left lung. However, he believes the nodule is medium risk for cancer based on its size, with a 5-15% risk of being cancer.*** *Nodules like this on people’s first CT are more suspicious and may pose a significant risk of cancer.*

*In disagreement with LungDetect AI,* ***Dr. Smith’s recommendation is to undergo an additional imaging test, called a PET/CT, now.*** *This would help us see how suspicious your nodule is. However, as mentioned before, the PET/CT has several downsides, such as exposure to higher radiation, and side effects, like fever and nausea.*

**
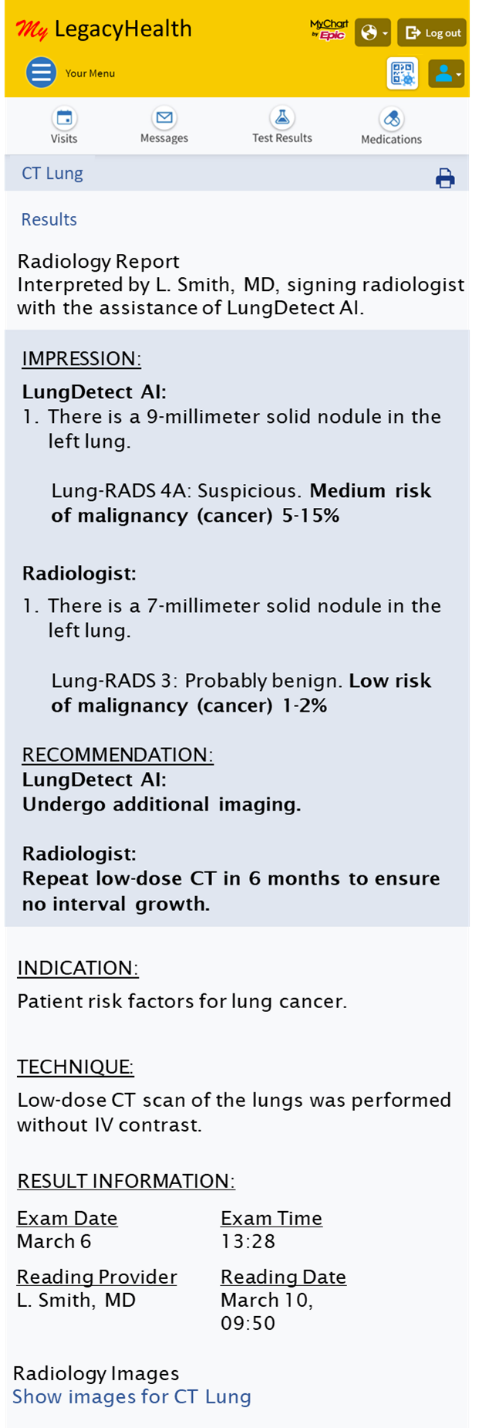
***Presented to participants in the radiologist-undercalls-AI condition*

Later that day, your PCP calls you to follow-up. He says:

*I wanted to call to follow-up. I saw you had your screening lung CT scan done last Wednesday and* ***the AI and radiologist are in disagreement.***

***Our LungDetect AI program identified a nodule with a medium risk of being cancer in your left lung.*** *The AI program is trained on thousands of low-dose CT images of lungs, both with and without cancer. Basically, the patterns on your scan match those in scans where the* ***patient ended up having cancer 5-15% of the time****.*

***LungDetect AI’s recommendation is to undergo an additional imaging test, called a PET/CT, now.*** *This would help us see how suspicious your nodule is. However, as mentioned before, the PET/CT has several downsides, such as exposure to higher radiation, and side effects, like fever and nausea.* ***Your reading radiologist, Dr. Smith, identified the same nodule in your left lung. However, he believes the nodule is low risk for cancer based on its size, with only a 1-2% risk of being cancer.*** *Nodules like this are common on people’s first CT and may remain stable over time and never pose a risk of cancer.*

*In disagreement with LungDetect AI,* ***Dr. Smith’s recommendation is to repeat the screening CT in 6 months.*** *This is to ensure the nodule is not growing. If the nodule remains stable, this will give us confidence that it is not cancer. If it does grow, we would recommend additional tests.*

*Presented to participants in each condition*

**Decision:**

Your PCP asks:

*Based on this recommendation, we can conduct a* ***follow-up screening CT in 6 months*** *or* ***additional testing (PET/CT) immediately.*** *Both will cost you the same amount. What do you want to do?*

1. Follow-up screening CT in 6 months
2. Additional testing (PET/CT)
